# Molecular epidemiological analysis of *Staphylococcus aureus* from hospital and food in Huai’an, Jiangsu, China, based on whole genome sequencing

**DOI:** 10.1038/s41598-026-49184-w

**Published:** 2026-04-20

**Authors:** Qihang Cai, Pengfei Yang, Bingbing Li, Qingli Yan, Fang He, Xiaohong Zhou, Hao Wang

**Affiliations:** 1 Lianshui County Center for Disease Control and Prevention, Lianshui, Huai’an, Jiangsu China; 2 Huai’an City Center for Disease Control and Prevention, Huai’an, Jiangsu China

**Keywords:** *Staphylococcus aureus*, MRSA, Mobile genetic elements, Biofilm, Pan-genomics, Phylogenetic analysis, Diseases, Genetics, Microbiology

## Abstract

*Staphylococcus aureus* (*S. aureus*) is a major pathogen causing severe infections and foodborne illnesses worldwide. Molecular epidemiological characteristics of *S. aureus* exhibit substantial temporal and regional differences. This study aims to investigate the genomic epidemiology of clinical (81 isolates) and food-derived (17 isolates) *S. aureus* strains from Huai’an, Jiangsu, China, during 2020 to 2021. Whole-genome sequencing revealed dominant multi-locus sequence typing (MLST) types, including ST398 (18.4%), ST1 (15.3%), and ST59 (11.2%). Hospital isolates showed higher methicillin-resistant *S. aureus* (MRSA) prevalence (58.0% vs. 41.2% in food), although the carriage rates of antimicrobial resistance (AMR) and virulence factor (VF) genes were similar between the two sources. Staphylococcal Chromosomal Cassette *mec* (SCC*mec*) type IVa(2B) (48.1%) predominated, suggesting community-associated MRSA infiltration into healthcare settings. Biofilm-associated gene profiles varied between MRSA and methicillin-susceptible *S. aureus* (MSSA), with ST1-t127 strains harboring comprehensive biofilm genes. Mobile genetic elements (MGEs), particularly plasmids (38.5%) and insertion sequences (32.3%), facilitated the dissemination of AMR and VF genes. Pan-genomic analysis demonstrated an open genome structure, with core genes enriched in metabolic functions (clusters of orthologous groups [COG] categories C, E, H, and P). Phylogenetic reconstruction revealed cross-regional transmission of ST1 and ST398, including international clades, while ST59 isolates exhibited clade diversification. This study elucidates the genetic diversity of *S. aureus* in Huai’an, Jiangsu, China, indicating its potential for cross-transmission between food and human sources and for regional dissemination, underscoring the need for One Health surveillance to curb the spread of high-risk clones.

**Supplementary Information:**

The online version contains supplementary material available at 10.1038/s41598-026-49184-w.

## Introduction

*Staphylococcus aureus* (*S. aureus*) is a versatile pathogen responsible for a wide range of infections, from minor skin conditions to life-threatening diseases such as sepsis and endocarditis^1^. Its significance is further magnified by its presence in both clinical settings and the food industry, where it poses a dual threat to public health^2^. The emergence of multidrug-resistant strains, particularly methicillin-resistant *S. aureus* (MRSA), has exacerbated the problem, leading to increased morbidity, mortality, and healthcare costs^3^. Besides, *S. aureus* on the surface of its host can contaminate animal food such as milk and meat, potentially causing staphylococcal intoxication^4^. Therefore, understanding the genetic diversity, virulence factors, and resistance mechanisms of *S. aureus* from both clinical and food sources is crucial for developing effective control strategies^5,6^.

The molecular epidemiology of *S. aureus* has been extensively studied using various typing methods. For instance, multi-locus sequence typing (MLST) and *spa* typing have been widely used to understand the population structure and evolutionary dynamics of *S. aureus*^7^. Similarly, staphylococcal chromosomal cassette *mec* (SCC*mec*) typing has been instrumental in identifying MRSA strains and their resistance profiles^8^. In addition to these classic methods for tracing the origin of clones and outbreaks, analyses of biofilm genes^9,10^, mobile genetic elements (MGEs)^11,12^, and pan-genome profiles can provide insights into the characteristics of prevalent strains in a region from different perspectives. On the one hand, MGEs act as key drivers of evolution by enabling horizontal gene transfer, dispersing critical traits such as virulence factors (VFs), antimicrobial resistance (AMR) genes, and other adaptive properties across populations^13,14^. On the other hand, biofilms help protect the bacterial cells from harmful extracellular environments such as antibiotics and the immune response of the host^15^. And pan-genomic analysis provides a macro perspective for understanding the overall genetic diversity of these strains^16,17^.

Given the significant regional distribution of *S. aureus*, molecular monitoring based on local data is crucial for understanding the transmission dynamics within communities and healthcare institutions, as well as identifying potential foodborne inputs. Huai’an, located in northern Jiangsu, is a major agricultural and food-processing hub with intensive livestock farming. According to official statistics, the annual output of pigs and poultry in Huai’an reached 2.69 million heads and 86.08 million birds, respectively, with a total meat production of 363,700 tons in 2024^18^. Previous local surveillance studies have revealed serious contamination of food products with *S. aureus*, with detection rates of 7.7% (18/233) in raw meat and poultry, and the isolated *S. aureus* strains exhibited high resistance to erythromycin (94.7%), trimethoprim/sulfamethoxazole (84.2%), and clindamycin (63.2%)^19^. Previous molecular epidemiological studies in Jiangsu have often relied on traditional typing methods (e.g., MLST, *spa* typing)^20,21^. However, no study in this region has yet integrated a comprehensive multi-perspective analysis that simultaneously examines traditional typing, biofilm gene profiles, MGEs, and pan-genomic structures. This is particularly true for Huai’an, where previous local surveillance has documented the presence of antimicrobial-resistant *S. aureus* in food products^19^, but systematic investigation of genetic links between clinical and food isolates remains absent. This integrated approach is essential for a ‘One Health’ surveillance strategy, which recognizes that human health is connected to the health of animals and the shared environment^22^. By identifying genetic links between food and clinical strains, we can track the movement of high-risk clones, such as community-associated MRSA (CA-MRSA) lineages—strains that originated in the community but are increasingly found in healthcare settings^23^.

In this study, we collected *S. aureus* strains from a tertiary hospital and food sources in Huai’an City, Jiangsu Province, China, and sequenced the whole genome. Then, the molecular characteristics, including MLST typing, *spa* typing, and SCC*mec* typing, as well as AMR and VF genes, were identified and compared. Biofilm-associated genes and MGEs in these strains were also detected. The analysis of pan-genomics was used to elucidate the core and accessory genomes of these *S. aureus* isolates from different sources. Finally, we analyzed the evolutionary epidemiology of the main prevalent strains (ST1, ST398, and ST59) among these *S. aureus* isolates and public data. These findings will provide us valuable insights into the pathogen’s evolution and transmission mechanisms, ultimately aiding in the development of targeted interventions in the local area.

## Results

### Sample composition

A total of 98 *S. aureus* isolates were included in this study (Supplementary Table S1). Eighty-one isolates were obtained from clinical specimens of patients at a tertiary hospital in Huai’an, comprising secretions (*n* = 43), sputum (*n* = 17), blood (*n* = 8), pus (*n* = 5), dialysis fluid (*n* = 4), ascites/pleural fluid (*n* = 2), urine (*n* = 1), and a renal vein catheter tip (*n* = 1). Seventeen isolates were collected from retail food markets, including poultry (chicken, *n* = 8; duck, *n* = 1), goat meat (*n* = 4), pork (*n* = 1), pig liver (*n* = 1), and *Monopterus albus* (*n* = 1).

### Phylogenetic analysis and molecular characteristics of 98 isolates

Core genome single nucleotide polymorphism (SNP)-based phylogenetic analysis showed a highly intermixed population structure, with clinical and food strains distributed throughout the tree without forming distinct host-specific clades (Fig. 2a). For instance, strains sp017, sp002, and sp004 showed high genetic similarity with cdc062, cdc054, and cdc017, respectively, with several pairs exhibiting near-zero genetic distances in either hospital or food groups.

**Fig. 1:**
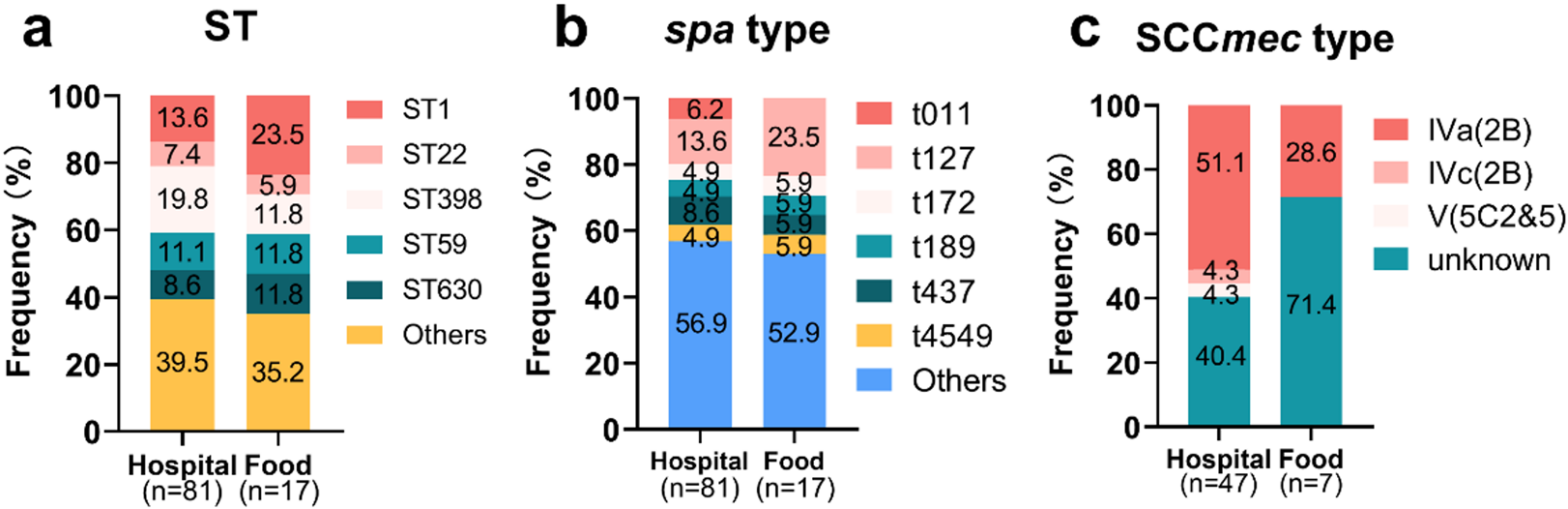
Distribution of S. aureus isolates of STs, spa types, and SCCmec types.

Among the 98 isolates, MLST successfully assigned sequence types to 89 isolates, identifying 18 STs, grouped into 6 clonal complexes (CCs). The distribution of predominant types differed between clinical and food sources (Fig. 1a). ST398 (19.8%, 16/81), ST1 (13.6%, 11/81), and ST59 (11.1%, 9/81) were the most common ST types in the hospital group. Several STs, including ST1281, ST15, ST30, ST4071, ST4513, ST5, ST6, ST8, and ST88, were exclusively identified in this group. ST1 (23.5%, 4/17), ST398 (11.8%, 2/17), and ST630 (11.8%, 2/17) were also detected from food, along with ST943 (11.8%, 2/17), which was exclusive to the food group.

**Fig. 2:**
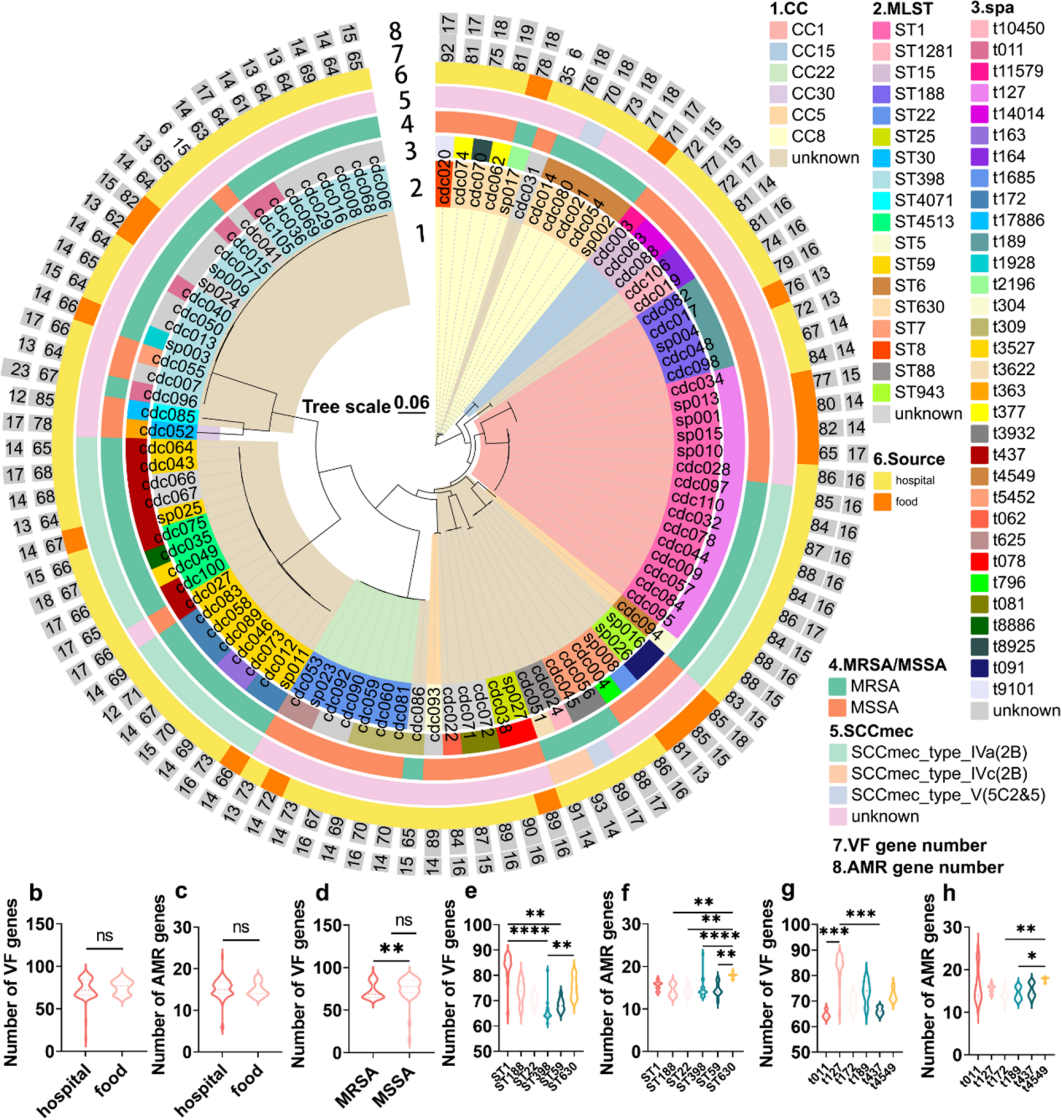
The approximate maximum-likelihood phylogenetic tree of 98 S. aureus isolates based on core genome SNPs and the distribution of molecular characteristics. (**a**) The approximate maximum-likelihood phylogenetic tree is shown in the center with clonal complexes (CC), MLST, spa types, the distribution of MRSA/MSSA, SCCmec types, VF genes, and AMR genes indicated by colors and mapped around the tree. (**b**) and (**c**) Comparison of the number of VF and AMR genes between hospital and food isolates. (**d**) Comparison of the number of VF genes between MRSA and MSSA isolates. (**e**) and (**f**) Comparisons of the number of VF and AMR genes among ST1, ST188, ST22, ST398, ST59, and ST630 isolates, respectively. (**g**) and (**h**) Comparison of the number of VF and AMR genes among t011, t127, t172, t189, t437, and t4549 isolates. “*” indicates *P* < 0.05, “**” indicates *P* < 0.01, “***” indicates *P* < 0.001, “****” indicates *P* < 0.0001, and “ns” indicates *P* > 0.05.

Similarly, among the 32 *spa* types identified successfully, there were 66 hospital isolates and 15 food isolates with various distributions (Figs. 1b and 2b). Notably, t127, t172, t189, t437, t4549, t625, and t078 were present both in hospital and food samples, accounting for 42.4% (28/66) of hospital isolates and 60.0% (9/15) of food isolates, respectively. Several other *spa* types were source-specific. Additionally, all t127 strains (100.0%, 15/15) were ST1, and 100.0% (5/5) of t189 isolates were associated with ST188. In contrast, some STs contained various *spa* types. More than half of ST 630 isolates were t4549 (55.6%, 5/9). Apart from undetectable *spa* types, t011 (60.3%, 5/8) was highly related to ST 398, although t1928, t2346, and t5452 were also identified in this ST type.

Hospital isolates held a higher proportion of MRSA strains than food counterparts, 58.0% (47/81) and 41.2% (7/17), respectively. In all sample types, the percentages of MRSA in secretion and dialysis fluid were more than half, which were 67.4% (29/43) and 75.0% (3/4). Based on MLST typing, ST59 (90.1%, 10/11), ST398 (88.9%, 16/18), ST1 (60.0%, 9/15), and ST630 (66.7%, 6/9) had a higher MRSA rate. Compared with MRSA, the diversity of STs of methicillin-susceptible *S. aureus* (MSSA) strains was greater, with 15 ST types. 54 MRSA strains were classified as IVa(2B) (48.1%, 26/54), IVc(2B) (5C2) (3.7%, 2/54), V(5C2&5) (3.7%, 2/54), and undetermined (44.4%, 24/54) (Figs. 1c and 2c). All undetermined isolates were confirmed to carry the *mecA* gene, confirming their MRSA status despite the lack of a definitive SCC*mec* type. Importantly, all IVc(2B) and V(5C2&5) strains were derived from secretion, although IVa(2B) accounted for approximately 32.6% (14/43) of this sample. Only two food isolates were successfully typed, both of which were classified as IVa(2B). In addition, ST1-IVa-t127 isolates (100%, 9/9) were all detected from hospital specimens.

The distribution of VF and AMR genes did not differ significantly between hospital and food isolates (Fig. 2b and c). Isolates from MSSA carried more VF genes compared with MRSA (Fig. 2d). STs with 5 or more strains were analyzed further. Figure 2e showed that ST1 isolates (*n* = 15, comprising 11 clinical and 4 food isolates) carried more VF genes than ST398 and ST630, and ST398 isolates (*n* = 18, 16 clinical and 2 food isolates) carried fewer VF genes than ST630. Figure 2f demonstrated that ST630 isolates (*n* = 9, 7 clinical and 2 food isolates) contained the most AMR genes except for ST1. Additionally, t127 isolates (*n* = 15, comprising 11 clinical and 4 food isolates) carried more VF genes than t011 and t437, while t4549 isolates (*n* = 5, comprising 4 clinical and 1 food isolates) exhibited a higher rate of AMR genes compared with t172 and t189 (Fig. 2g and h). Differential analysis of individual genes revealed that hospital isolates carried significantly more toxin genes, including *sea* (encoding staphylococcal enterotoxin A), *selk*, and *selq* (superantigen toxins), compared to food isolates (*P* < 0.05). In contrast, food isolates exhibited a significantly higher carriage rate of the adhesion gene *clfB* (*P* < 0.05), which mediates binding to host tissues and may facilitate colonization on food matrices (Supplementary Table S2). Similarly, aside from the *ermC* gene, which confers resistance to macrolides via ribosomal methylation, there were no significant differences in AMR genes between hospital and food isolates (Supplementary Table S3).

### Biofilm-associated gene distribution

**Fig. 3:**
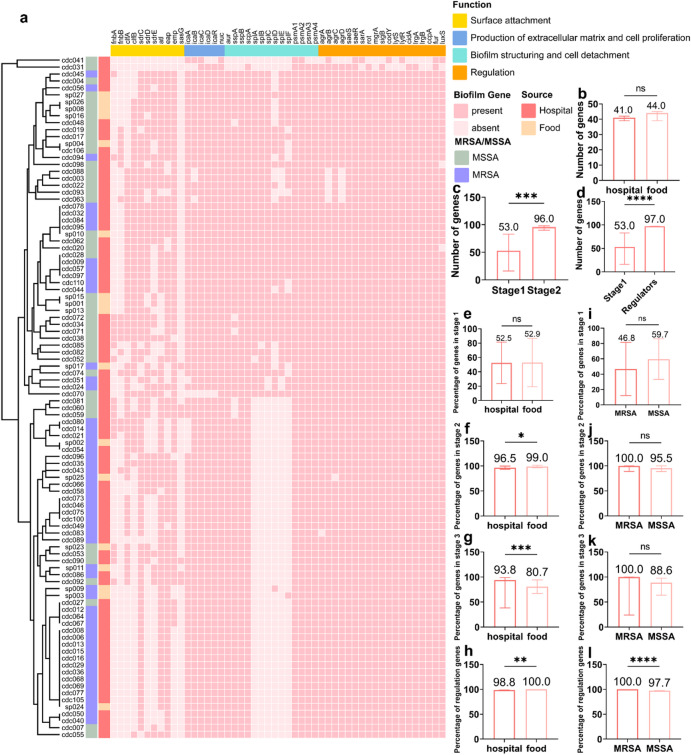
Characteristics of the distribution of biofilm-associated genes. (**a**) Binary heatmap of 50 genes corresponding to 3 stages of biofilm formation and regulation. The X-axis shows 50 biofilm-associated genes classified by different stages. The Y-axis represents the clustering of 98 isolates based on Euclidean distance and classified by source and MRSA/MSSA distribution. (**b**) Comparison of biofilm gene numbers between hospital and food isolates. **(c)** Comparison of biofilm gene numbers between stage 1 (surface attachment) and stage 2 (production of extracellular matrix and cell proliferation). (**d**) Comparison of biofilm gene number between stage 1 and the regulation process. (**e**–**h**) Comparisons of genes in different stages or related to regulation between hospital and food isolates, respectively. (**i**–**l**) Comparisons of genes in different stages or related to regulation between MRSA and MSSA, respectively. “^*^” indicates *P* < 0.05, “^**^” indicates *P* < 0.01, “^***^” indicates *P* < 0.001, and “^ns^” indicates *P* > 0.05.

The distribution of biofilm-associated genes is shown in Fig. 3a. A total of 50 genes related to biofilm were classified into four sections in this study, including 11 genes in the surface attachment stage (stage 1), 6 genes in the production of extracellular matrix and cell proliferation stage (stage 2), 14 genes in the biofilm structuring and detachment stage (stage 3), and 19 genes associated with different regulating processes (regulators). The number of these genes carried by hospital isolates was comparable to that of food strains (Fig. 3b). Detection rates of genes in the first stage were significantly lower than those in stage 2 and the regulators (Fig. 3c and d). As shown in Fig. 3e–h, the percentages of biofilm-associated genes in stage 2, stage 3, and the regulation process were higher in the food group. Based on single-gene analysis, there were no differences in detection rates of hospital and food isolates (*P* > 0.05, Supplementary Table S4).

Fig. 3i–l demonstrated that the percentages of regulatory genes in MRSA were higher than those in MSSA. MRSA isolates exhibited significantly higher carriage rates of genes including *atl* (encoding autolysin, which mediates cell lysis and extracellular DNA release during biofilm maturation), *icaB* (involved in polysaccharide intercellular adhesin synthesis), and the accessory gene regulator (agr) quorum sensing system genes (*agrB*, *agrD*). In contrast, MSSA isolates carried higher frequencies of genes encoding microbial surface components recognizing adhesive matrix molecules (MSCRAMMs), such as *fnbA*/*fnbB* (fibronectin-binding proteins), *clfB* (clumping factor B), and *sdrD*/*sdrE* (serine-aspartate repeat proteins), which are critical for primary attachment to host extracellular matrix proteins. Among food isolates, MSSA carried significantly higher frequencies of serine protease genes (*splA*-*C*, *splE*, *splF*) compared to MRSA. All *P* values of the above analysis were lower than 0.05, and the data are provided in Supplementary Table S4.

Excluding genes with a carriage rate greater than 90%, other genes were used to analyze their correlations with main STs (Supplementary Table S5). All ST1 strains (*n* = 15) contained *clfA*, *clfB*, *eap*, *emp*, and *splA*-*C* and *E* without *sasG*. All ST398 isolates (*n* = 18) carried *eap* and lacked *fnbA*, *fnbB*, *emp*, *sasG*, and *splA-C* and *E*. All ST59 (*n* = 11) isolates did not carry *fnbA*, *clfB*, *sasG*, and *splA*-*F* but carried *eap* and *emp*.

### Mobile genetic elements prediction

**Fig. 4:**
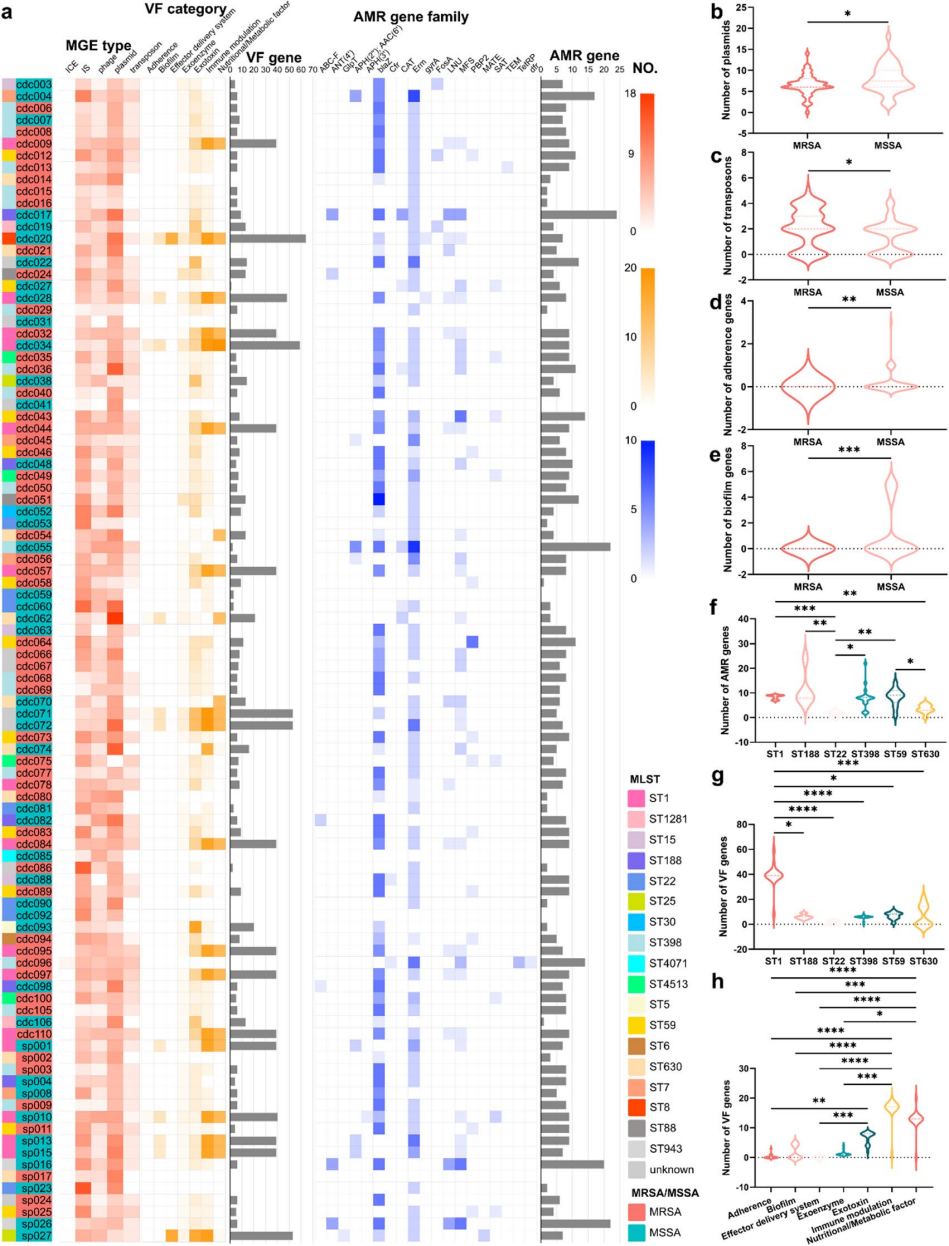
Distributional characteristics of MGEs and their carriage of VF and AMR genes. (**a**) Distribution of MGEs, VF categories, VF gene number, AMR gene families, and AMR gene number. MLST and MRSA/MSSA were colored. (**b**–**e**) Comparisons of the numbers of plasmids, transposons, adherence genes, and biofilm genes between MRSA and MSSA isolates, respectively. (**f**) and (**g**) Comparisons of AMR and VF gene number among ST1, ST188, ST22, ST398, ST59, and ST630 isolates, respectively. (**h**) Comparisons of VF gene numbers across seven functional categories: Adherence genes mediating attachment to host cells or extracellular matrix; Biofilm genes directly involved in biofilm formation; Effector delivery system genes encoding secretion systems or effectors; Exoenzyme genes encoding secreted enzymes; Exotoxin genes encoding toxins; Immune modulation genes involved in evading host immune responses; Nutritional/Metabolic factor genes facilitating nutrient acquisition and metabolism within the host. “^*^” indicates *P* < 0.05, “^**^” indicates *P* < 0.01, “^***^” indicates *P* < 0.001, and “^****^” indicates *P* < 0.0001.

In total, 1,829 MGEs were predicted in 98 isolates, including plasmid (38.5%, 705/1,829), insertion sequence (IS, 32.3%, 590/1,829), phage (19.5%, 356/1,829), transposon (9.7%, 177/1,829), and integrative and conjugative element (ICE, 0.1%, 1/1,829). Prediction of VF and AMR genes associated with MGEs identified 1,289 VF genes and 688 AMR genes, respectively (Fig. 4a). Among the 688 MGE-associated AMR genes, the majority were carried by plasmids (504 genes, 73.3%), followed by unit transposons (84 genes, 12.2%), transposons (79 genes, 11.5%), phages (15 genes, 2.2%), and IS (6 genes, 0.9%). For VF genes (1,289 in total), plasmids also dominated (742 genes, 57.6%), with a substantial contribution from phages (547 genes, 42.4%). No VF genes were detected in association with IS, transposons, or unit transposons in our dataset. Similar to the whole genome results, there were no significant differences in all VF and AMR genes carried by MGEs between hospital and food *S. aureus* isolates (Supplementary Tables S6 and S7).

To understand the correlation between MGEs and strain types, sample source, *mecA* carriage, and STs (STs with 5 or more strains were included in the statistics) were analyzed in relation to MGEs prediction data. The number of plasmids in MSSA was significantly higher than in MRSA (Fig. 4b), while MRSA carried a relatively higher number of transposons (Fig. 4c). Adherence and biofilm genes were both more frequently detected in MSSA than in MRSA, as shown in Fig. 4d and e. The relationship between MLST and AMR genes demonstrated that ST22 isolates carried fewer AMR genes than other STs except for ST630 (Fig. 4f). In addition, ST1 strains carried a higher proportion of VF genes than all other ST *S. aureus* strains, which showed no statistically significant differences among them (Fig. 4g). Further analysis of the relationship among VF categories in ST1 strains showed that exotoxin, immune modulation, and nutritional/metabolic factors were the main VF gene classes (Fig. 4h).

### Pan-genome analysis

**Fig. 5:**
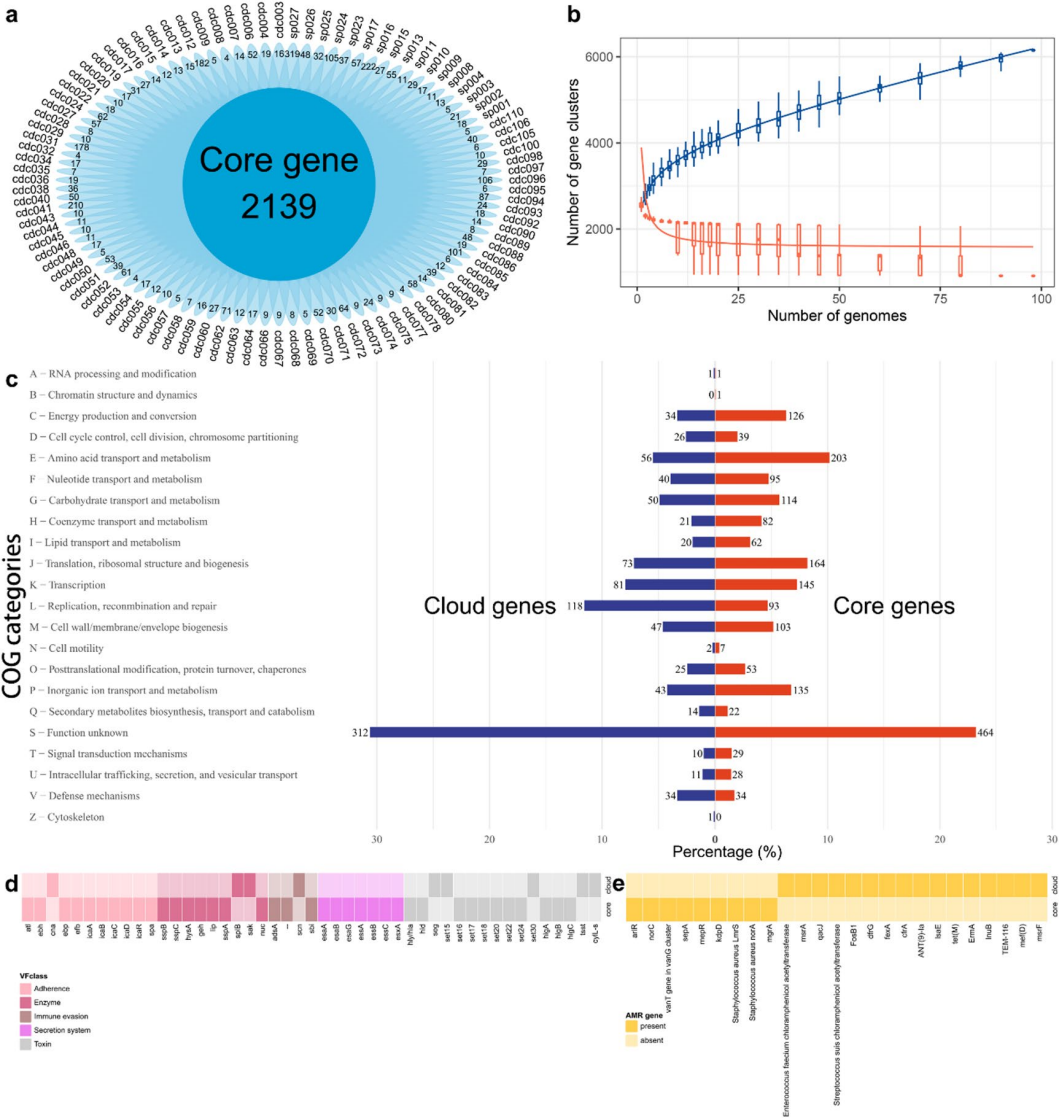
Pan-genome analysis of 98 *S. aureus* isolates. (**a**) Distribution of core and cloud genes in each genome. (**b**) Core-pan rarefaction curve. (**c**) COG classification of core and cloud genes. (**d**) Binary heatmap showing the distribution of virulence genes between core and cloud genomes and VF class with different colors. (**e**) Distribution of AMR genes between core and cloud genomes.

To further dissect the pan-genome structure, genes were categorized into three groups based on their prevalence across the 98 isolates: core genes (present in < 95% of genomes, *n* = 2,139), accessory genes (present in 5–95% of genomes, *n* = 1,377), and cloud genes (present in < 5% of genomes, mean = 35.08 ± 50.34 per genome) (Fig. 5a). It is evident that the pan-genome curve of these isolates in this study increased with the increase of the number of detected strains, while the core genome size remained stable (Fig. 5b). This pattern indicates that these isolates possess an open pangenome. Based on clusters of orthologous groups (COG) annotation shown as Figs. 5c and 2000 core genes and 1019 cloud genes were classified into 22 categories. C (energy production and metabolism), E (amino acid transport and metabolism), H (coenzyme transport and metabolism), and P (inorganic ion transport and metabolism) genes were mainly located in the core genome, while L (replication, recombination, and repair), S (function unknown), and V (defense mechanisms) processes were significantly associated with the cloud genome (*P* < 0.05, Supplementary Table S8). Figure 5d and **e** demonstrated that the core genome contained more VF genes, while a higher level of AMR genes was present in the cloud genome.

To identify genes potentially associated with niche adaptation, we compared the distribution of accessory genes between clinical and food isolates. A total of 54 genes were exclusive to clinical isolates and 1 gene exclusive to a food isolate (Supplementary Table S10). Among the 37 clinical-specific genes with functional annotations, several encode virulence factors (e.g., coagulase, staphylococcal toxin, agr system components), mobile genetic element proteins (integrases, recombinases, transposases), regulatory elements (MarR, toxin-antitoxin systems), and metabolic/transport functions. The sole food-specific gene encodes a DDE-domain transposase, suggesting a mobile element of unknown significance. The imbalance likely reflects the larger clinical sample size and greater genetic diversity in healthcare settings.

### Phylogenetic analysis of ST1, ST398, and ST59

**Fig. 6:**
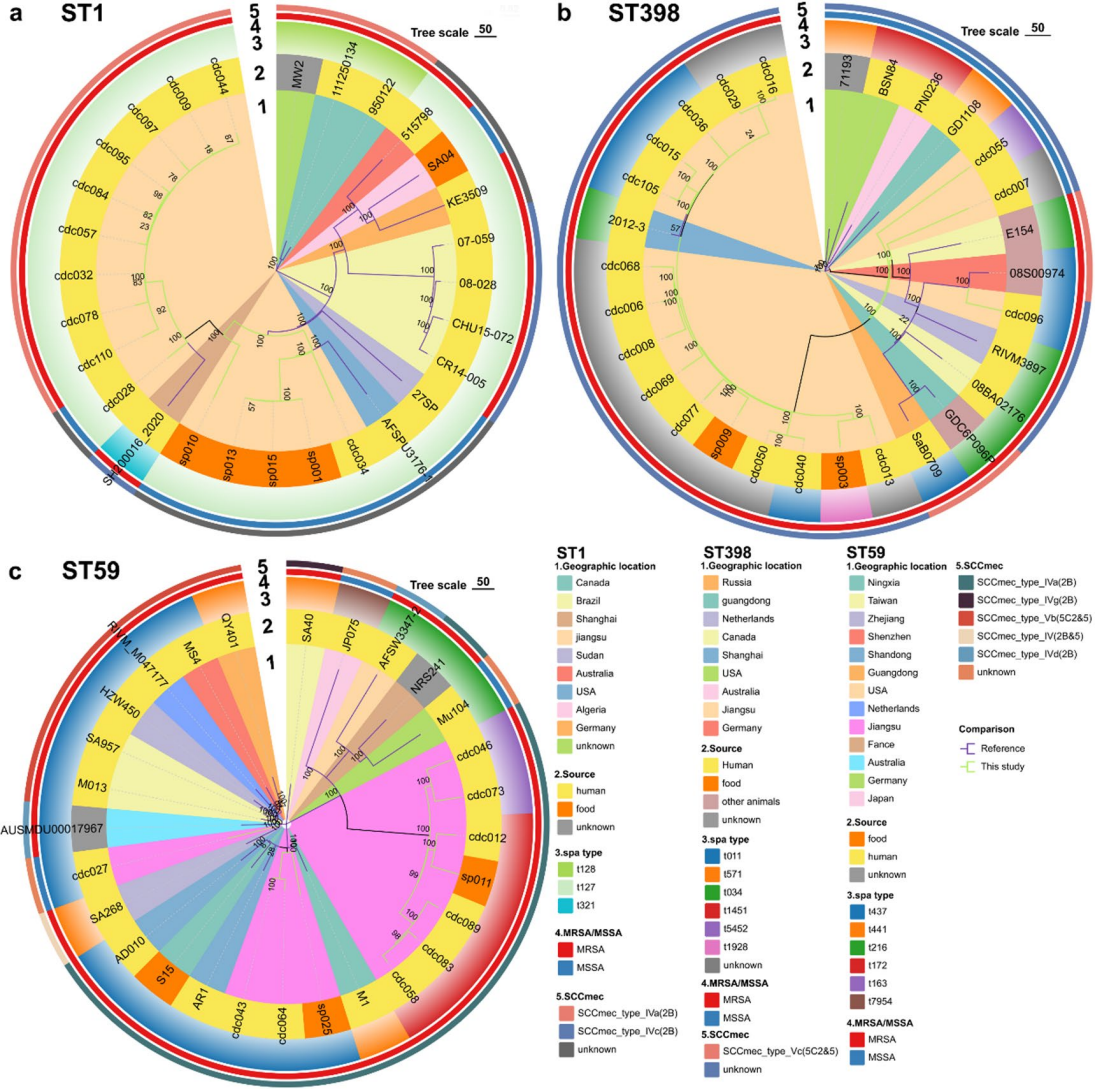
Reconstructed phylogenetic trees of ST1s, ST398s, and ST59s based on core SNPs. Information on geographic location, sample source, *spa* type, MRSA/MSSA, and SCC*mec* was mapped with different colors. (**a**) Phylogenetic tree of ST1s including 15 isolates in this study and 13 genomes from the NCBI database. MW2 was used as the reference strain. (**b**) Phylogenetic tree of ST398s, including 18 isolates in this study and 11 genomes from the NCBI database. 71,193 was used as the reference strain. (**c**) Phylogenetic tree of ST59s, including 11 isolates in this study and 17 genomes from the NCBI database. SA40 was used as the reference strain.

To further reveal the prevalence of 3 major STs in this study, *S. aureus* genomes from different geological regions were collected and phylogenetic trees were reconstructed. As shown in Fig. 6a, all ST1 isolates in this study were t127 and in a clade. Most hospital strains were associated with SH200016_2020, a human isolate from Shanghai (bootstrap = 100%), and the genetic distance between SH200016_2020 and the closely related human isolate (cdc028) and food isolate (sp010) were 196.0 SNPs and 350.3 SNPs, respectively. Our food isolates (sp001, sp015, sp013) and the clinical isolate cdc034 clustered with AFSPU3176-1 from a human in the USA (100% bootstrap support), with pairwise genetic distances to AFSPU3176-1 ranging from 255.7 to 283.0 SNPs.

Most ST398 strains in our research showed close correlation to a Shanghai human isolate (2012-3), and all of them were MRSA (Fig. 6b). Our human isolate cdc096 was associated with the Germany animal strain (bootstrap = 100%, 112.9 SNPs), both of which were t011. And cdc007 as well as cdc055 also had a long genetic distance from other isolates in this study.

ST59 isolates in this study were distributed into three well-supported phylogenetic clades (Fig. 6c). The first clade (bootstrap = 100%) included cdc043, cdc064, and sp025, clustering with domestic reference strains (Ningxia and Shanghai isolates). The second clade (bootstrap = 100%) comprised human isolates (cdc073, cdc046, cdc012, cdc089, cdc058, and cdc083) and the food isolate (sp011), grouping with international reference human strains (NRS241 from France, Mu104 from Germany, AFSW3347-2 from the USA, and JP075 from Japan), although the genetic distance between our strain and foreign strains is at least 448.2 SNPs. The remaining isolate, cdc027, formed a separate clade (bootstrap = 100%) with several national isolates and other international references, suggesting a mixed lineage. The genetic distance between cdc027 and SA268, a human isolate from Zhejiang, was 205.8 SNPs.

## Discussion

In this study, we analyzed the molecular epidemiological characteristics of 98 *S. aureus* isolates obtained from the hospital (*n* = 81) and food (*n* = 17) in Huai’an, Jiangsu Province, eastern China. Our analysis provides critical insights into strain source, molecular typing, MGEs, biofilm gene profile, genome structure, and potential transmission routes.

To explore the potential for cross-transmission between different niches, we began by characterizing the strain population structure. The higher MRSA prevalence in hospital isolates (58.0% vs. 41.2% in food) aligns with the role of healthcare environments in fostering antibiotic resistance. The dominance of IVa(2B) (48.1% of MRSA) suggests CA-MRSA lineages infiltrating hospitals, as type IV is typically linked to community transmission^23^. The high proportion (44.4%) of MRSA isolates with undetermined SCC*mec* types likely reflects the known structural heterogeneity of SCC*mec* elements. Recombination events, novel combinations of *ccr* and *mec* complexes, or truncated *ccr* genes can prevent classification by standard typing assays. This finding underscores the genetic diversity of SCC*mec* in our region and suggests that some isolates may carry novel or hybrid variants, warranting further characterization using long-read sequencing technologies. Importantly, the absence of a definitive SCC*mec* type does not diminish their clinical significance as MRSA, as all carried the *mecA* gene. Crucially, we identified 18 distinct ST types, with ST398 (18.4%), ST1 (15.3%), and ST59 (11.2%) being the most prevalent. The co-occurrence of *spa* types (t127, t172, t189, t437, t4549, t625, and t078) in both hospital and food isolates raised concerns about potential cross-contamination risks.

Multiple lines of evidence support the possibility of cross‑transmission between humans and food in Huai’an. First, the identification of identical molecular types in both hospital and food isolates, specifically ST1‑t127, ST398, ST59‑t172, and ST630-t4549, provides a strong genetic fingerprint of overlapping strain populations. This sharing of *spa* and ST types suggests a common source or frequent exchange between these compartments. The high prevalence of livestock‑associated ST398 in both clinical and food isolates also raises the possibility of farm‑to‑fork transmission via the food supply chain^24^. Second, phylogenetic analysis revealed close genetic relationships between clinical and food isolates within the same ST lineages. For example, ST1 food isolates (sp001, sp010, sp013, and sp015) and clinical isolates (cdc034, cdc028, cdc097, etc.) clustered in a clade, suggesting recent common ancestry or potential direct transmission. This close clustering indicates recent common ancestry and is consistent with either direct transmission or exposure to a common contamination source. Contaminated food products, particularly raw or undercooked meat, may serve as vehicles introducing *S. aureus* into households and healthcare settings^25^. Conversely, occupational exposure among food handlers could facilitate bidirectional spread, with colonized individuals contaminating food during processing^26^. Third, consistent with the whole-genome analysis, no significant differences were observed in the carriage of individual AMR or VF genes on MGEs between hospital and food isolates, which further implies horizontal gene transfer across niches. While our genomic data reveal close genetic relationships between clinical and food isolates, these findings should be interpreted as suggestive evidence of potential epidemiological links rather than conclusive proof of transmission.

The observed capacity for cross-niche transmission is consistent with the genomic characteristics of the *S. aureus* population in this study. The open pan-genome structure observed in our study underscores the remarkable genomic plasticity of these pathogens. The COG annotation revealed that core genes dominated metabolic processes (COG categories C, E, H, and P), and cloud genes were enriched in regulatory and environmental response functions (L and V). This mirrors the evolutionary strategy of *S. aureus*—preserving core metabolic networks for host colonization while dynamically acquiring accessory genes to exploit novel environments^27^. The compartmentalization of VF and AMR genes between the core and cloud genome highlights the mobility and variability of these genes. This inherent genomic flexibility enables the pathogen to rapidly adapt to selective pressures encountered in different niches. The striking imbalance in the number of source‑specific genes (54 vs. 1) is likely influenced by the larger sample size of clinical isolates and the greater genetic diversity typically encountered in clinical settings due to antibiotic selection and host interactions. These observations underscore the need for integrated One Health surveillance across human, animal, and food compartments to disrupt transmission pathways^22^.

The distinct biofilm gene profiles of MRSA and MSSA suggest divergent biofilm formation strategies with implications for niche adaptation. MRSA isolates showed enrichment of PIA‑dependent biofilm genes (*icaB*) and the agr quorum sensing system (*agrB*, *agrD*). PIA‑dependent biofilms are particularly advantageous in clinical environments, where they promote adherence to medical devices, protect against antibiotics, and facilitate immune evasion, although this type of biofilm was mainly observed for MSSA^28^. Studies have demonstrated that the introduction of *mecA* into MSSA strains can repress PIA production and attenuate PIA-mediated biofilm formation. Therefore, the *icaB* genes we detected may largely represent a “silent” genetic archive. The MRSA isolates, characterized by high *atl* prevalence and elevated agr expression, present a genotype suited for immune evasion and dissemination and might be considered less suited to long-term persistence on food contact surfaces. A biofilm that is constantly dispersing is less effective as a stable surface contaminant. However, the dispersed single cells or small clusters resulting from agr activity could themselves be contaminants, leading to sporadic contamination events. Conversely, MSSA isolates carried higher frequencies of MSCRAMM genes (*fnbA*, *fnbB*, *clfB*, *sdrD*/*E*), which mediate direct attachment to host extracellular matrix proteins. This protein‑mediated adhesion may favor survival in food‑related niches, such as on meat surfaces or food‑processing equipment, where initial colonization is critical and antibiotic pressure is lower. Based on clonal lineage-specific gene repertoires, our research illustrates that the conserved biofilm gene profile of ST1-t127 strains mirrors their success as cross-regional pathogens. In contrast, ST1, ST398s, and ST59s lacked various biofilm-associated genes to some extent. The overall similarity of biofilm genes between clinical and food isolates, with subtle, type-specific differences, may be associated with microenvironment-specific selective pressures^29^. Further functional studies are needed to confirm these genotype-phenotype associations.

MGE analysis further supports niche‑specific adaptation. Our MGE annotation identified plasmids (38.5%) and IS (32.3%), which respectively disseminate AMR genes horizontally and potentially accelerate resistance evolution^30^. Plasmids carried the majority of both AMR (73.3%) and VF (57.6%) genes, confirming their primary role as vehicles for horizontal gene transfer in staphylococci. Phages contributed substantially to VF carriage (42.4%) but played a minimal role in AMR dissemination (2.2%), consistent with their known function in mobilizing virulence factors via lysogenic conversion. Transposons and unit transposons collectively accounted for 23.7% of AMR genes, indicating their involvement in resistance gene mobilization, often through composite transposon structures. The negligible AMR gene load on insertion sequences (0.9%) suggests they primarily facilitate gene capture and rearrangement rather than direct resistance carriage. These findings highlight the complementary roles of plasmids (dominant for both AMR and VF), phages (specialized for VF), and transposons (auxiliary for AMR) in shaping the adaptive potential of *S. aureus* in this region. Although there was no statistical difference in the number of the AMR gene at the genome level, ST22 isolates exhibited the lowest AMR gene burden in MGE results, which is consistent with monitoring results in Malaysia^31^. MRSA compared to MSSA carried significantly higher transposon and lower plasmid abundance. In contrast, adherence and biofilm genes were predominantly found in MSSA. These results may reflect the preference of different genes for transmission vectors. High transposon levels may implicate long-term genomic stability and multidrug resistance, while low plasmid levels reduce horizontal gene transfer (HGT) in the short term. This likely contributes to the persistence of MRSA’s multidrug resistance phenotype. Once a resistance gene is integrated into the chromosome via transposons, it is less likely to be lost (plasmid curing) compared to strains that carry resistance only on plasmids. This may partly explain why MRSA infections are often associated with more aggressive and prolonged therapeutic requirements.

Over recent decades, the ST1-IV lineage has been globally associated with community-acquired infections in various nations^32–35^. The predominance of *spa* type t127 and its exclusive linkage to ST1 reflect a conserved evolutionary relationship, as observed in Greece MRSA outbreaks^36^. ST1 isolates in this study clustered within a clade containing both clinical and food-derived strains (Fig. 6a). Notably, the close phylogenetic relationship between hospital isolates (our and Shanghai isolates) and food strains (our and USA isolates) suggests potential cross-contamination pathways. ST1 strains carried the highest VF gene load, dominated by exotoxins, immune modulators, and metabolic factors. This comprehensive virulence repertoire likely underpins the success of ST1 as a cross‑host pathogen. This observation, along with our MGE prediction, aligns with reports of ST1-t127 as a “generalist” lineage capable of colonizing both humans and livestock, facilitated by MGEs encoding antibiotic resistance and adhesion factors^37^. As has been reported, ST1-SCC*mec*IV strains have evolved to thrive in hospital environments by attenuating their virulence while enhancing their capacity for persistence and intracellular survival within host cells. Such adaptive strategies may help offset the biological cost associated with maintaining multiple antibiotic resistance genes^38^. Notably, ST1‑t127 has been associated with severe infections, including fatal pediatric sepsis and necrotizing pneumonia in the US^33,39^, invasive hospital infections in Brazil^35^, and multidrug-resistant outbreaks with bloodstream infections in Ireland^40^. The presence of food isolates clustering with a U.S. reference strain raises concerns about international food trade as a vector for *S. aureus* dissemination (Fig. 6a).

The predominance of ST398 (18.4%) across all isolates aligns with previous reports of their prevalence in Tianjin, Northern China^41^. In contrast to reports from Europe, where human‑adapted ST398 is predominantly MSSA, ST398 in this study exhibited a strong association with MRSA (88.9%)^42,43^. This discrepancy may reflect regional differences in antibiotic use in livestock. In China, intensive farming practices involve heavy antimicrobial use, selecting for MRSA ST398 in pigs and poultry, which can then spill over into humans through occupational exposure or the food chain^44–47^. The clustering of most Huai’an ST398 isolates with Shanghai hospital MRSA strains (Fig. 6b) suggests regional dissemination within healthcare networks, while the presence of a strain related to a German pig isolate (cdc096) points to potential international introduction via livestock trade^48,49^. The long genetic distance of strains like cdc007 and cdc055 suggests localized diversification, potentially driven by antibiotic selection or niche-specific adaptation in closed hospital units^50^.

ST59 of CA-MRSA has emerged as the dominant lineage across the Asia-Pacific region, exhibiting substantial phylogenetic heterogeneity^51^. It was one of the major MRSA lineages in China, accounting for 14.9%, and has been shown to exhibit diminished resistance to most tested antimicrobial agents and harbor fewer genetic determinants of resistance^52^. The phylogenetic diversification of ST59 isolates into three distinct clades (domestic, international, and mixed; Fig. 6c) reflects the ecological plasticity of this lineage. The domestic clade (including cdc043, cdc064, and sp025) clustered with strains from Ningxia and Shandong, indicating interprovincial dissemination of ST59 within China, likely through human movement or livestock trade. The international clade grouped with strains from diverse geographic origins (USA, Japan, Europe), indicating global dissemination of this lineage, likely through travel or trade. The mixed clade containing cdc027 may represent a recombinant or intermediate lineage, possibly arising from horizontal gene transfer between different ST59 subpopulations. This clade diversification may reflect adaptation to different niches (hospitals, farms, or food environments) driven by acquisition of niche‑specific mobile elements and resistance genes.

Several limitations should be considered when interpreting our findings. First, this study is limited by its single-center retrospective design with a small sample size, lacking detailed patient demographic and food geographic and temporal information, and its focus on analysis at the draft genome level, and therefore precludes definitive conclusions about transmission directionality or specific routes. Second, this study relied solely on genomic data without corresponding phenotypic validation (e.g., biofilm formation ability, antibiotic susceptibility testing). A multicenter study with a large sample size can provide better guidance on the prevalence of *S. aureus* in Huai’an. Long-read sequencing could resolve MGE-mediated gene transfer networks, including VF and AMR genes. Future studies should integrate genomics with phenomics to confirm the functional relevance of biofilm and AMR genes.

Based on our findings, several targeted interventions and research priorities emerge. First, enhanced surveillance of high‑risk clones (ST1‑t127, ST398, and ST59) in the food chain should be implemented. Second, strengthening infection control measures and promoting rational antibiotic use, particularly targeting beta‑lactams and macrolides, could help curb MRSA transmission and reduce selective pressure for resistant clones. Third, coordinated surveillance across sectors, such as longitudinal sampling of livestock, retail meat, farm workers, and hospital patients, would clarify transmission dynamics and identify intervention points. Fourth, future work should explore whether these genomic features translate into enhanced virulence (e.g., in animal models) or differential environmental persistence and whether they are linked to mobile genetic elements that could be targeted to disrupt dissemination.

## Conclusion

Our results showed that there is a possibility of food and human cross-transmission as well as internal and external dissemination of *S. aureus* in the Huai’an area. The overall levels of VF and AMR genes are similar between hospital and food isolates, though this is an open pan-genome with type-specific expression of biofilm-associated genes and MGEs. Major STs (ST1, ST398, and ST59) present the dual capacity for regional transmission and cross-host adaptation. ST1 strains carried the highest VF gene load, including the most comprehensive biofilm-associated genes. ST398 exhibited a strong association with MRSA, which may be related to the current emergence of this type in China, especially in Jiangsu. ST59, as the dominant lineage across the Asia-Pacific region, remains prevalent in Jiangsu. These results partially reflect molecular epidemiology features of Huai’an and may aid in the regional prevention and control of *S. aureus* strains in a One Health background.

## Materials and methods

### Isolates collection

Clinical isolates were obtained from the Chinese Pathogen Identification Net, which receives submissions from a tertiary hospital in Huai’an, Jiangsu, China, between October 2020 and October 2021. All isolates were collected as part of routine clinical diagnostics and pathogen surveillance. The hospital laboratory staff collected clinical specimens from patients during standard care and isolated *S. aureus* from these specimens. Only the fully anonymized bacterial isolates (with all patient identifiers removed) were submitted to our center for genomic analysis. The specimen types of these isolates are summarized in the Results section (Sample composition). As this study involved only bacterial isolates from routine surveillance and did not involve human subjects, human tissues, or identifiable personal data, ethical approval was not applicable. All methods were carried out in accordance with relevant national guidelines and regulations.

Food-derived isolates were obtained through the routine food safety surveillance program of the Huai’an City Center for Disease Control and Prevention. As part of ongoing surveillance activities, fresh, chilled, or frozen types of food samples were purchased quarterly from markets or shops of different counties and districts. The sampling covered a variety of food types (poultry, red meat, offal, aquatic products) but was not formally stratified by food category or market type, as the samples were originally collected for routine monitoring rather than for a predefined research protocol. Within each sampling period, vendors were selected randomly to ensure a degree of representativeness, although the sampling was not formally stratified by food category or market type. After collection, samples were placed in sterile bags and transported to the laboratory at 4 °C. Samples were stored at 4 °C for no more than 24 h to minimize post-collection contamination and bacterial overgrowth. All *S. aureus* strains were confirmed through the VITEK 2 Compact Fully Automatic Biochemical Identification Device and kept frozen at − 80 °C.

The genomes reported here were successfully sequenced from the available viable isolates.

### Whole-genome sequencing

The whole genome shotgun (WGS) strategy was used to construct libraries with 400 bp inserted fragments, and the libraries were sequenced by next-generation sequencing (NGS) based on the Illumina NovaSeq platform. The sequencing achieved an average depth of 633.0× (range: 179.9× to 1301.2×), with > 99.9% genome coverage and a Q30 score > 91.7% for all samples, ensuring high-quality data for downstream analyses. The adapter sequences and low-quality sequences were removed for clean reads using fastp 0.20.1^53^. All data were assembled using SPAdes 3.15.3^54^. The resulting assemblies were then subjected to contamination screening using GenBank Foreign Contamination Screen (FCS) automatically and removing manually based on the FCS reports before submission^55^.

### Bioinformatic analysis

The assembled data were applied for MLST typing via the MLST database^56^, *spa* typing via *spa*Typer 1.0^57^, and MRSA and SCC*mec* typing via SCCmecFinder 1.2^58^.

To identify biofilm-associated genes, we curated a list of 50 *S. aureus* genes known to be involved in biofilm formation based on the comprehensive review by Schilcher and Horswill^9^ and the primary studies cited therein. Genes were identified by matching product names from Bakta annotations (Software: 1.11.0, Database: 6.0.0)^59^, and each candidate gene was then manually verified based on its functional description to confirm its role in biofilm formation processes. The full list of these 50 genes is provided in Supplementary Table S4.

Pan genome analysis was performed via IPGA v1.09^60^ using default parameters. COG classification of core and cloud genes was acquired from the eggNOG-mapper 2.1.12 result^61,62^.

VF and AMR genes were acquired via sequence blast with VFDB^63^ and CARD^64^ databases, respectively. For VF genes, we used the default parameters of the VFDB web server. For AMR genes, the Resistance Gene Identifier (RGI) tool was used with the CARD database under the following settings: Perfect, Strict, and Loose algorithms, with filtering for complete genes only, and the 95% identity nudge option enabled.

MGE analysis was performed via the MGEs and Transferable ARGs and VFs detection tool^65^, which integrates multiple software packages for comprehensive MGE annotation^66^. The following tools and parameters were employed: ISEScan (v1.7.2.3, --remove Short IS, *evalue* ≤ 1e-5), DANMEL (v2.13.0+, Identity > 90%, Scoverage > 80%), MobileElementFinder (v1.1.1, Coverage > 80%, *evalue* ≤ 1e-5, Identity > 90%, Scoverage > 80%), ICEFinder (v1.0, *evalue* ≤ 1e-5, Identity > 90%, Scoverage > 80%), BacAnt (v3.4.0, Identity > 90%, Scoverage > 80%), IntegronFinder2.0 (v2.0.2, --local-max --func-annot), Platon (v1.6, --verbose), PlasmidFinder (v2.1.6.1, Identity > 90%, Scoverage > 80%).

### Phylogenetic analysis

Approximate maximum-likelihood phylogenetic (AML) trees of all 98 isolates in this study were constructed using core genome SNPs via Parsnp 2.1.3 with default parameters. For the phylogenetic analysis of major STs (ST1, ST398, ST59), publicly available *S. aureus* genomes were retrieved from the NCBI database (Supplementary Table S9). Reference genomes were selected based on geographic representation to contextualize our local strains within global phylogenetic diversity, prioritizing complete genomes or high-quality draft assemblies, and confirmation of sequence type (ST) using the same MLST typing tool as for our isolates (see “Bioinformatic analysis” section). For each of these STs, the core genome alignments generated by Parsnp were subjected to recombination detection and removal using Gubbins v3.4.3^58^. Gubbins was run with the following parameters: --tree-builder raxml (using RAxML for tree reconstruction) and --bootstrap 1000 (to obtain branch support values).

### Statistical analysis and graphical drawing

The data passing normality and homogeneity of variance tests used unpaired or paired *t-tests* for comparing two groups and one-way ANOVA for comparing multiple groups; the data failing to pass the normality or homogeneity of variance tests used the Mann-Whitney test for comparing two groups and the Kruskal-Wallis test for comparing multiple groups, followed by Dunn’s multiple comparisons test. Dunn’s test applies a Bonferroni correction to adjust for multiple testing. Fisher’s exact test was used for frequency data analysis. Chart drawing used GraphPad Prism 9. *P* values of < 0.05 were reported as statistically significant.

Heatmaps and phylogenetic trees were visualized using ChipPlot^67^. R ggplot2 was used for generating part of the images.

## Electronic Supplementary Material

Below is the link to the electronic supplementary material.

Supplementary Material 1

## Data Availability

The sequencing data generated in this study have been deposited in the NCBI databases under BioProject accession number PRJNA1233917 (https://www.ncbi.nlm.nih.gov/bioproject/PRJNA1233917).
